# Domestic Summary

**Published:** 1839-06-01

**Authors:** 


### DOMESTIC SUMMARY.

### PHILADELPHIA DISPENSARY.

*Reports for April*, 1839.

Dr. Patterson has treated thirty-two cases in the northeast district. Of these, there were two cases of pneumonia; one of pleuritis; six of catarrh; three of pertusses; one of hypochondriasis; one of phthisis; two of delirium tremens; one of marasmus; one of hæmorrhoids; six of scarlatina; one of ophthalmia; three of hysteria; two of fever, (one of intermittent, one of remittent;) one of diarrhœa; one of gastritis—thirty-two. Twenty-nine of these patients recovered, two were relieved, and one died.

Dr. Boyer has treated thirty-eight cases in the north middle district. Of these, there were two cases of bronchitis; one of catarrh; one of contusion; three of cynanche tonsillaris; three of diarrhœa; two of dysentery; one of emesis; two of erysipelas; two of remittent fever; one of chronic laryngitis; one of menorrhagia; two of ophthalmia; one of otitis; one of pertussis; one of pericarditis; one of pleurodynia; two of phthisis; three of rheumatism; four of scarlatina; one of scirrhous uteri; three of irritation from dentition. Thirty-two of these patients recovered, two were relieved, two removed out of the district, and two died.

Dr. Boyer has also prescribed for seventythree cases at the consultation office of the dispensary. The results of these cases are not well ascertained.

Dr. Knight has treated seventeen cases in the southeastern district. Of these, there were one case of chronic gastritis; one of pleuritis; one of neuralgia; one of rheumatism; two of dysentery; two of remitting fever; two of intermittent fever; one of bronchitis; one of ascites; one of sprain; one of ophthalmia; one of croup; one of catarrh; one of herpes—seventeen. Of these, eleven have been cured, four remain under cure, one relieved, and one discharged for non-compliance with prescriptions.

Deaths from scarlatina, one; chronic laryngitis, one; phthisis, one: three out of eighty-seven cases.

In the south middle district, from the 15th to the 31st of April, Dr. Berkeley has treated fourteen cases, viz.: one case of cholera, (a black sailor,) cured by the use of grain doses of calomel and opium every hour until relieved. Two cases of dropsy—one, in an infant born with œdema of the face and extremities, gradually extending over the body, with effusion into the abdominal cavity; died. The second, a woman, (black,) aged twenty-eight years, found in a dying state at the first visit, in consequence of exhausting diarrhœa, attendant on ascites of several months' duration. Three cases of rheumatism, cured; three cases of diarrhœa, in children, the result of intestinal irritation from worms, cured; one case of leucorrhœa, cured; one of pneumonia, consequent on asthma of long standing, cured; two of phthisis, not terminated,—and one of remittent fever, cured.

---

*Extraordinary Tendency to the Formation of Urinary Calculi.* By THOMAS SEWALL, M. D.—Salvador Catalano, aged 72, a native of Italy, came to this country in 1805, in the capacity of sailing master. Soon after his arrival he was employed by our government, in the Navy Yard at Washington, as an inspector of ordnance. He had hitherto possessed a robust and vigorous constitution, and a remarkable exemption from bodily infirmity. About twenty years since, while at work in the yard, he received a severe blow over the loins from an iron bar. Immediately after the accident he felt a desire to void urine, and upon making the effort, discharged a considerable quantity of this fluid, mixed with blood. This was followed by severe pain in the region of the kidneys, attended with symptomatic fever; but after a few weeks' confinement, he was so far relieved as to return to his accustomed occupation. From this time he suffered more or less from weakness and stiffness of the loins, and from pain in the region of the kidneys, ureters and bladder, which occasionally extended down the thighs to the knees, and when most severe was followed by a swelling of the testes.

About four years from the time of the accident, having suffered for some days from an attack of acute pain travelling down in the direction of the left ureter, he discovered in his urine a number of calculi, about the eighth of an inch in diameter, ragged and angular. From this time he was induced to inspect his urine as it was discharged, and to filtrate it, and found that it yielded from a half to a full teaspoon of calculi daily. For one or two years he preserved the calculi thus collected, placing the product of each day in an apartment by itself. Within a few days I have examined his cabinet of calculi, consisting of several hundred parcels, which he preserves as a curiosity, and values above all price. From the slight examination I made, I should judge that each parcel contained from twenty grains to two drachms. They were obtained by first filtrating the urine, and then washing the residuum in several waters, so that a considerable portion of the finer part of the concretions were washed away, as he told me, with the dregs.

This patient still continues to discharge about the same amount of calculi as formerly, and from the account which he gave me, and from the specimens in his possession, I should suppose that the kidneys must have elaborated not less than one drachm daily for the last fifteen years, amounting in all to a little more than forty-five pounds.

I have had no opportunity of ascertaining the chemical composition of this formation, as no analysis has been made; but judging from appearance, there is a difference in the constitution of the portions voided at different times, as they are somewhat various in color, as well as in texture and size.

For some years the habits of Catalano have been sedentary. He lives mostly on vegetable food, with a small proportion of animal broth, and takes a few glasses of gin and water daily. He also drinks freely of melon and flax-seed tea, and also elm and other mucilaginous diuretic preparations. But the article from which he derives the most benefit, is the Harlem oil, which he takes in doses of twenty-five drops every other night. This medicine, he assures me, not only acts as a diuretic, but prevents the formation of calculi of large size, and that when he takes it the most freely the concretions are voided in the form of sand.—*Bost. Med. Jour.*

